# Effect of Solution Temperature on Corrosion Behavior of Ti-6Al-4Zr-3Nb-1.1Mo-1Sn-1V Alloy in Hydrochloric Acid Solution

**DOI:** 10.3390/ma19010047

**Published:** 2025-12-22

**Authors:** Chengliang Mao, Siyuan Zhang, Silan Li, Jialu Wang, Qian Li, Weiju Jia

**Affiliations:** Northwest Institute for Nonferrous Metal Research, Xi’an 710016, China

**Keywords:** titanium alloy, microstructure, electrochemical techniques, corrosion behavior

## Abstract

Ti-6Al-4Zr-3Nb-1.1Mo-1Sn-1V (Ti90) alloy is widely used in marine engineering and oil and gas extraction due to its excellent strength, impact toughness, and corrosion resistance. The corrosion behavior of Ti90 alloy after solution treatment at 750 °C, 900 °C, 940 °C, and 960 °C in 5 M hydrochloric acid (HCl) solution was investigated using open-circuit potential (OCP), potentiodynamic polarization, electrochemical impedance spectroscopy (EIS), static immersion tests, and surface characterization. The results of electrochemical tests indicate that the corrosion resistance of Ti90 alloy increases with rising solid solution temperature. The static immersion tests show that the variation trend of the annual corrosion rate at different solid solution temperatures in 5 M HCl solution is consistent with the electrochemical test results. The corrosion morphology of Ti90 alloy reveals that the α phase is more prone to decomposition than the β phase. The corrosion behavior of Ti90 alloy in 5 M HCl solution is mainly influenced by the volume fraction of the β phase and the size of the α phase.

## 1. Introduction

Titanium alloy has high corrosion resistance and is often used in harsh service environments. The surface of titanium alloy contains a dense passivation film mainly composed of TiO_2_, which contributes not only to corrosion resistance but also to biocompatibility [1,2,3,4,5]. This oxide film exhibits strong corrosion resistance in most service environments. In some cases, even if damaged, it can repair itself rapidly. However, the passivation film on titanium alloy faces a relatively high risk of damage in reducing acid solutions. According to the existing literature, the main factors influencing the corrosion behavior of titanium alloys include alloy composition, microstructure characteristics, and usage environment [6,7,8,9,10].

Microstructure plays a significant role in the corrosion behavior of titanium alloy [2,11]. Titanium alloys subjected to different thermomechanical processing and heat treatment processes exhibit substantial microstructural differences, mainly involving morphological features, grain size, phase content, and even dislocation density [12,13]. The influence of different microstructures on the corrosion resistance of titanium alloys is intricate and complex, leading to inconsistent conclusions among researchers. Wei et al. [14]. investigated the corrosion and passivation behavior of Ti-3Mo alloy in 20 wt.% HCl solution at solution temperatures ranging from 750 °C to 1050 °C. The corrosion rate increased with rising treatment temperature, which was attributed to accelerated corrosion of the passivation film above 850 °C and the establishment of micro-galvanic cells at the α/β phase interface. Meng et al. [15]. studied the corrosion resistance of Ti80 alloy annealed from 800 °C to 1040 °C in 5 wt.% HCl solution. As the treatment temperature increased from 800 °C to 960 °C, the corrosion resistance improved. When the annealing temperature reached 1040 °C, the corrosion rate decreased to the minimum value. The variations in α and β phase fractions and grain size were identified as the main factors affecting the corrosion resistance of Ti80. An increase in β-phase fraction and grain refinement enhanced the corrosion resistance. Currently, research on the microstructure of titanium alloys primarily focuses on its influence on mechanical properties, while the effect of microstructural differences on corrosion behavior has not been extensively studied. Therefore, this work aims to investigate the effect of microstructural variations under different solution treatment temperatures on the corrosion behavior of Ti90 alloy.

Service condition is another important factor affecting the corrosion resistance of titanium alloy. Titanium alloys exhibit excellent corrosion resistance due to their ability to form stable passivation films under most working conditions. However, these films are prone to decomposition in concentrated reducing acid solutions (HCl, H_2_SO_4_), resulting in relatively poor corrosion resistance in such environments [16,17,18,19,20,21]. Fracturing technology is a critical step in oil and gas extraction. During this process, acidic substances such as hydrochloric acid are typically injected into the well, creating a downhole environment rich in H^+^ and Cl^−^ ions. Therefore, it is of great significance to study the corrosion behavior and mechanism of titanium alloys in HCl solution. Ti90 alloy was developed based on TC4 titanium alloy by adding appropriate amounts of Mo, Zr, Nb, V, and Sn. As a near-α titanium alloy, Ti90 exhibits outstanding corrosion resistance and mechanical properties, with a yield strength exceeding 900 MPa. This alloy is commonly used for manufacturing seamless titanium tubes serving in oilfield fluids rich in H^+^ and Cl^−^. Since the relationship between microstructure and corrosion characteristics of Ti90 alloy has not yet been established, this work aims to investigate the corrosion resistance of Ti90 alloy with different microstructures in hydrochloric acid solution. Furthermore, the specific mechanism by which microstructural factors influence corrosion behavior was examined, providing essential corrosion data to support the application of Ti90 alloy.

## 2. Material and Methods

Ti90 alloy ingot was produced by melting sponge titanium and master alloys in a vacuum consumable arc furnace. The ingot was processed through billet opening and reforging to form an 80 mm thick slab. Subsequently, the Ti90 alloy was rolled in multiple passes within the two-phase zone, reducing the thickness from 80 mm to 12 mm. The α/β phase transition temperature of the Ti90 alloy sheet was 965 °C. The measured chemical composition of Ti90 alloy is presented in Table 1.

The Ti90 alloy sheet billet was subjected to solid solution treatment in an electric heating furnace at 750 °C, 900 °C, 940 °C, and 960 °C, respectively. The holding time was 1 h, followed by air cooling. The heat-treated plates were cut by electrical discharge wire cutting to prepare metallographic specimens (10 × 10 × 5 mm), electrochemical specimens (10 × 10 × 3 mm), and static immersion specimens (20 × 10 × 4 mm). All samples were taken along the lengthwise direction of the Ti90 sheet.

The metallographic specimens were prepared as follows: Ti90 alloy specimens were sequentially ground using silicon carbide (SiC) papers of 150#, 800#, and 1000# grit sizes, and then polished to a mirror finish with a silica suspension. The samples were subsequently washed with water and alcohol, etched with Kroll’s reagent (87 mL H_2_O, 9 mL HF, and 4 mL HNO_3_) for 3–5 s, and finally dried by blowing [22]. The microstructure of the Ti90 alloy was examined using VHX-2000 optical microscopy (OM) (Keyence, Osaka, Japan) and IT510 scanning electron microscopy (SEM) (JEOL, Akishima City, Tokyo, Japan), while the distribution of alloying elements was analyzed with an IT510 energy dispersive spectrometer. The size and content of phases in the microstructure were measured using Image Pro Plus (IPP) software (Version 6.0). Phase analysis of uncorroded metallographic specimens was performed using X-ray diffraction (XRD, Bruker D8) (Bruker AXS GmbH, Karlsruhe, Germany) with a Cu Kα radiation source (wavelength = 1.5418 Å) operating at 40 kV and 40 mA.

Given the need to mimic the actual corrosive environment of oil and gas wells, a 5 M HCl solution was therefore chosen as the corrosive medium in this study. The electrochemical tests were performed using a Metrohm PGSTAT 302N (Metrohm Autolab, Utrecht, the Netherlands) electrochemical workstation. The test temperature was maintained at 30 ± 1 °C. A conventional three-electrode system was employed, with the sample as the working electrode, a platinum sheet as the counter electrode, and a saturated silver chloride electrode as the reference electrode. The electrochemical samples were prepared as follows: Copper wires were welded onto the back of the samples, which were then embedded in E-44 epoxy resin to expose a surface area of 1 cm^2^. After the epoxy resin solidified, the samples were ground sequentially with 150#, 800#, and 1000# grit SiC sandpaper, polished to a mirror finish using a silica suspension, ultrasonically cleaned with deionized water and alcohol, and finally blow-dried. The electrochemical testing procedure consisted of three stages: first, the open-circuit potential (OCP) was measured until stabilization; second, electrochemical impedance spectroscopy (EIS) was performed with a 10 mV peak-to-peak potential perturbation, scanning from 10^5^ Hz to 10^−2^ Hz; finally, potentiodynamic polarization curves were recorded by scanning from −0.25 V to 2.5 V (relative to OCP) at a rate of 0.001 V/s. Each sample underwent three repeated tests following this procedure to ensure data reliability.

The corrosion resistance of Ti90 alloy was evaluated through static immersion experiments. The samples were sequentially ground with 150#, 600#, and 1000# grit SiC sandpaper, polished to a mirror finish using a silica suspension, ultrasonically cleaned with deionized water and alcohol, and then dried by blowing. The mass of each sample was determined using an analytical balance with an accuracy of 0.001 g, and all samples were immersed in a beaker containing 5 M HCl solution. During immersion, the beakers were maintained in a water bath at 30 ± 1 °C for 7 days. After soaking, the samples were ultrasonically cleaned in acetone, deionized water, and alcohol for 10 min each, dried, and reweighed. Three parallel samples were tested for each condition. The corrosion morphology after immersion was examined using scanning electron microscopy (SEM).

## 3. Results and Discussion

### 3.1. Microstructure Evaluation

**Figure 1:**
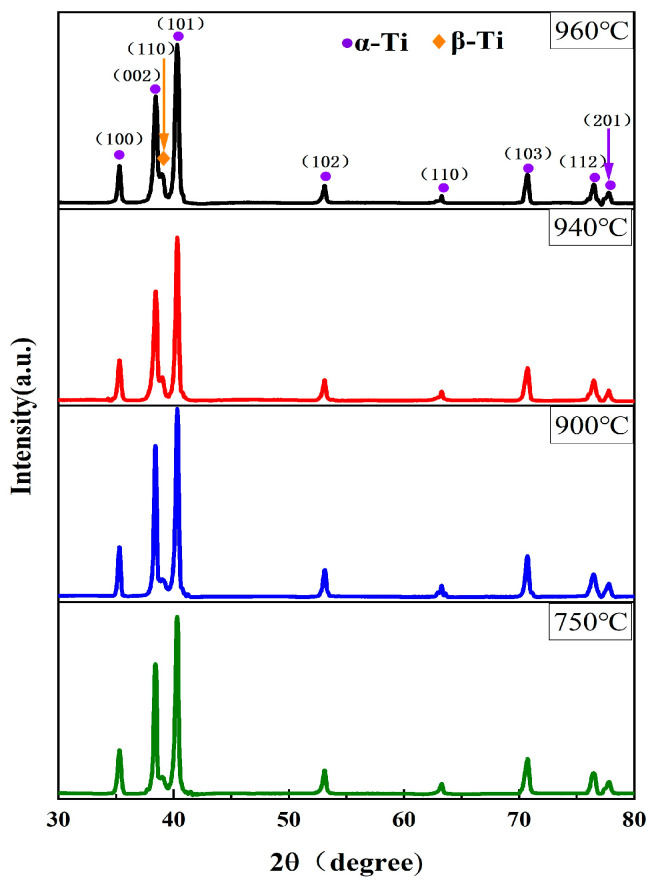
The XRD patterns of Ti90 alloy treated at different solution temperatures.

Figure 1 shows the XRD patterns of Ti90 alloy samples after solid solution heat treatment. All solution-treated Ti90 samples consist of α phase with a hexagonal close-packed (hcp) structure and β phase with a body-centered cubic (bcc) structure. As the solution temperature increases, the β phase reflection peak (approximately 2θ = 38.5°) shifts and its intensity rises with increasing solution temperature. These observations qualitatively indicate that the β phase content in Ti90 alloy gradually increases with rising solution temperature.

**Figure 2:**
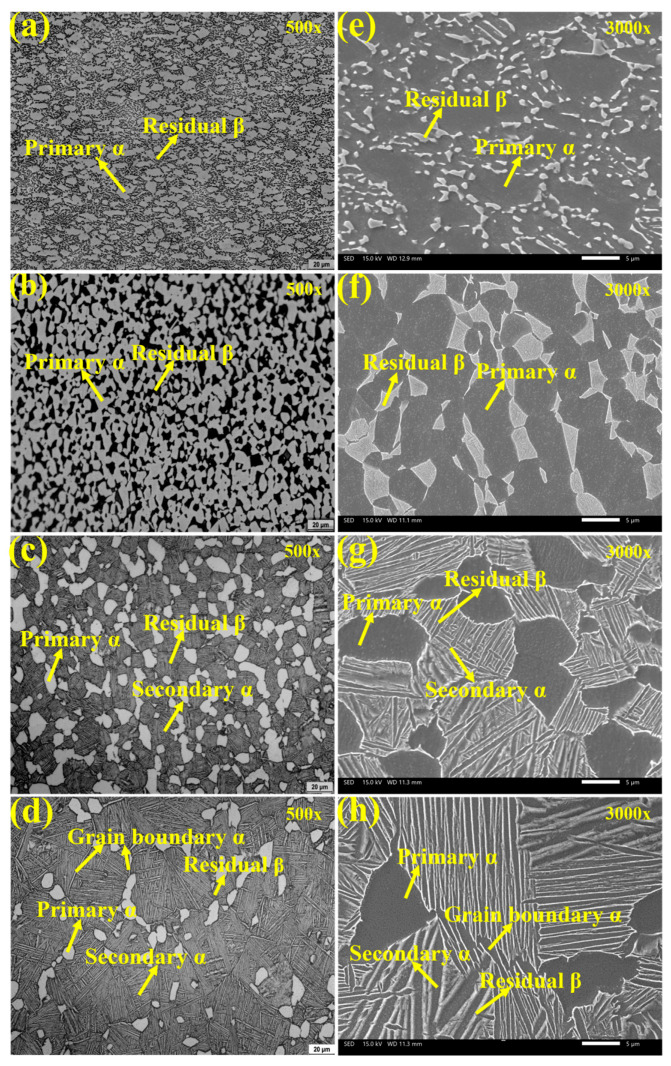
The microstructure of Ti90 alloys treated at different solution temperatures, as shown by OM (**a**–**d**) and SEM (**e**–**h**): (**a**,**e**) 750 °C; (**b**,**f**) 900 °C; (**c**,**g**) 940 °C; and (**d**,**h**) 960 °C.

**Figure 3:**
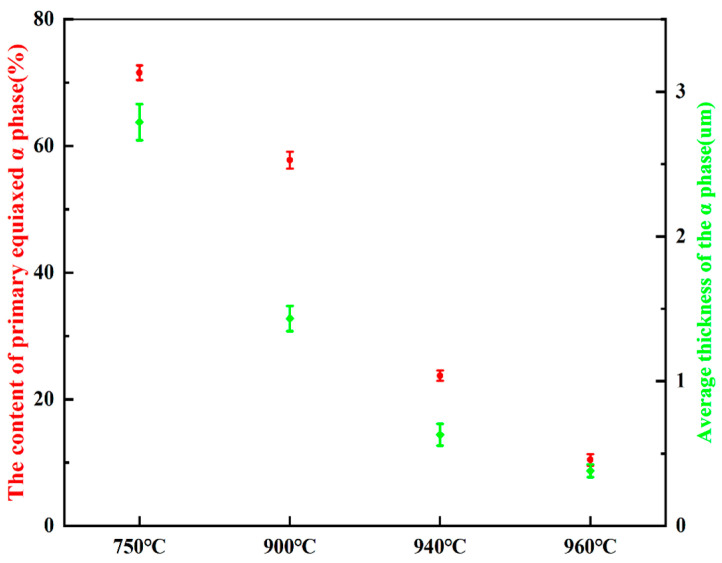
The content of primary α phase and the average thickness of α phase in the microstructure of Ti90 alloys treated at different solution temperatures.

Figure 2a–h depict the microstructure of Ti90 alloys in the solution-treated condition. As shown in Figure 2a,e, the samples treated at 750 °C consist primarily of primary equiaxed α phase and a small amount of residual β phase. The significant amount of primary equiaxed α phase is attributed to incomplete phase transformation during annealing, caused by the relatively low solution temperature and short holding time. As seen in Figure 2b,f, when the solution temperature was raised to 900 °C, the volume fraction of primary equiaxed α phase decreased, while the proportion of secondary lamellar α phase increased, resulting in a reduction in the average α phase thickness. Figure 2c,g show that after solution treatment at 940 °C, the content of primary equiaxed α phase further decreased, whereas the residual β phase content increased. The amount of secondary lamellar α phase distributed within the residual β phase also rose, further reducing the average α phase thickness. At 960 °C (in the vicinity of the α/β transition point), the content of primary equiaxed α-phase reached a minimum, while α-phase precipitated along the original grain boundaries. As shown in Figure 2d,h, after solution treatment at 960 °C, the residual β phase content was the highest and the α phase thickness was the smallest. As summarized in Figure 3, statistical analysis using IPP software (Version 6.0) indicates that with increasing solution temperature, the primary α phase content decreases from 71.5% to 10.4%, and the average α phase size decreases from 2.79 μm to 0.38 μm.

### 3.2. Electrochemical Analysis

**Figure 4:**
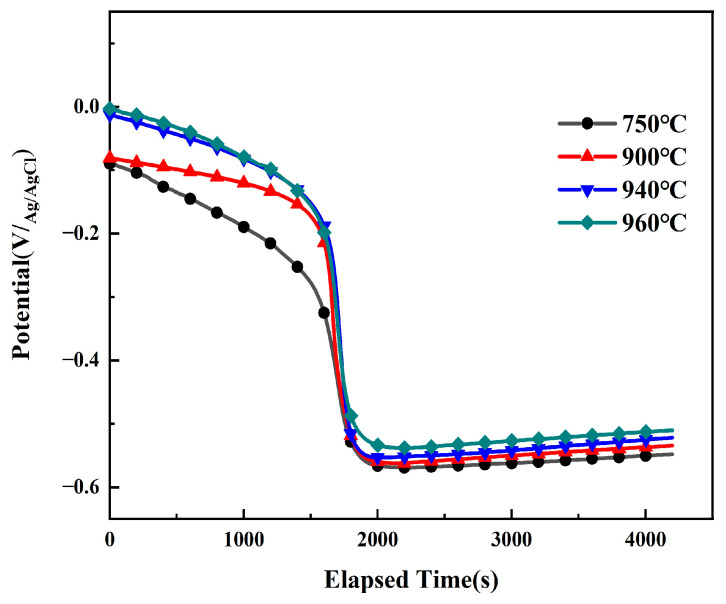
The OCP of Ti90 alloys treated at different solution temperatures in 5 M HCl solution.

Figure 4 shows the OCP curves of the solution-treated Ti90 alloy immersed in 5 M HCl solution for 4200 s. The OCP parameters represent the thermodynamic tendency of electrochemical oxidation for Ti90 titanium alloy in HCl medium. During the initial immersion stage, the potential of all samples first shifted slowly in the negative direction and then dropped sharply to a relatively low value. This negative shift may be attributed to localized damage of the primary oxide film on the titanium alloy surface in the early stage of immersion. With prolonged immersion time, the OCP gradually shifted toward more positive values, indicating the occurrence of repassivation or an increase in the thickness of the passivation film in the solution [23,24]. Eventually, the OCP stabilized, and the metal/solution interface reached a steady state. A more positive open-circuit potential (stable OCP value) corresponds to a higher corrosion resistance of the material. As shown in Figure 4, the stable OCP values of Ti90 samples gradually increased with rising solution temperature, indicating enhanced corrosion resistance of Ti90 alloy at higher solution temperatures.

**Figure 5:**
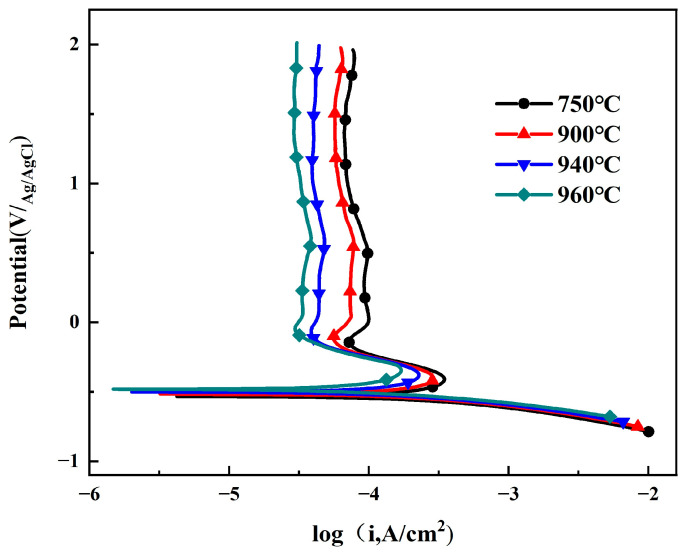
The polarization curves of Ti90 alloys treated at different solution temperatures in 5 M HCl solution.

Figure 5 shows the potentiodynamic polarization curves of Ti90 alloy after different solution treatments in 5 M HCl solution. The polarization curves at different solution temperatures exhibit similar shapes, indicating comparable electrochemical reactions on the electrode surface: the cathodic reaction corresponds to hydrogen evolution [25], while the anodic reaction comprises the active dissolution region, active–passive transition region, and passive region [26]. In the active dissolution region, as the potential shifts positively, the current density (*i*_corr_) increases rapidly, likely due to the breakdown of the primary passivation film, leading to active dissolution of the alloy. With further positive potential shift, the increase in *i*_corr_ is suppressed, which may be attributed to the formation of a new protective oxide film on the surface. When the potential exceeds 0 V, the anodic branch enters the stable passive region, where the formation and dissolution of the oxide film reach equilibrium, and *i*_corr_ stabilizes, consistent with the OCP test results [27]. A linear Tafel region is observed on the anodic side of the polarization curves. The corrosion potential (*E*_corr_) and corrosion current density (*i*_corr_) were determined by Tafel extrapolation of the cathodic branch. The current density at +0.5 V vs. Ag/AgCl was taken as the passivation current density (*i*_pass_). All polarization parameters are summarized in Table 2. The results show that with increasing solution treatment temperature, *E*_corr_ shifts positively, while both *i*_corr_ and *i*_pass_ gradually decrease. This demonstrates that the corrosion resistance and passivation film stability of Ti90 alloy improve with higher solution treatment temperatures.

**Figure 6:**
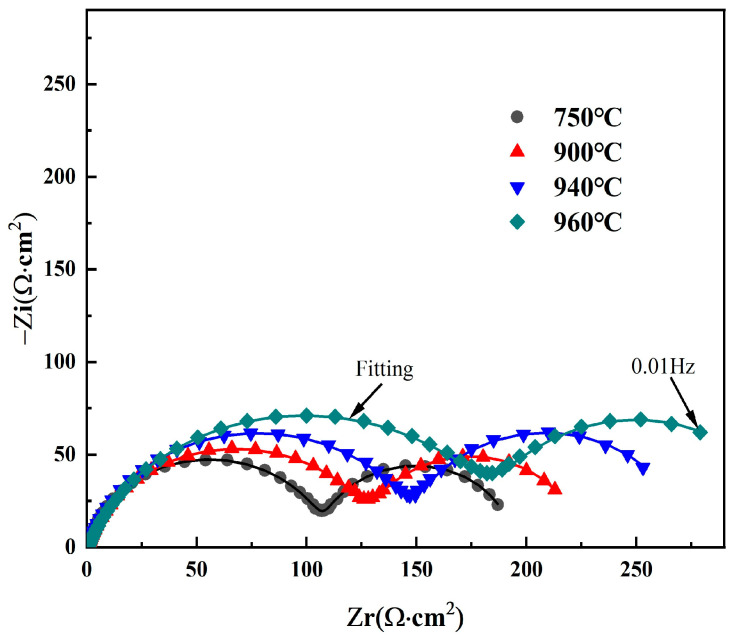
Nyquist diagrams of Ti90 alloy treated at different solution temperatures in 5 M HCl solution.

To further clarify the influence of different solution temperatures on the corrosion resistance of Ti90 alloy, electrochemical impedance spectra were measured in 5 M HCl solution. The corresponding Nyquist and Bode plots are shown in Figure 6. All Nyquist plots display two distinct semicircular arcs, indicating the presence of two capacitive time constants [28,29]. This also demonstrates that the corrosion mechanism remains unchanged despite variations in solution temperature. The high-frequency capacitive loop is attributed to the electrical double layer at the electrolyte interface, while the low-frequency capacitive loop is associated with the dissolution of the surface passivation film. Moreover, as the solution temperature increases from 750 °C to 960 °C, the diameters of both the high-frequency and low-frequency semicircular arcs increase. This indicates that raising the solution treatment temperature enhances both the charge transfer resistance and the corrosion product film resistance, thereby improving the corrosion resistance of Ti90 alloy in 5 M HCl solution.

**Figure 7:**
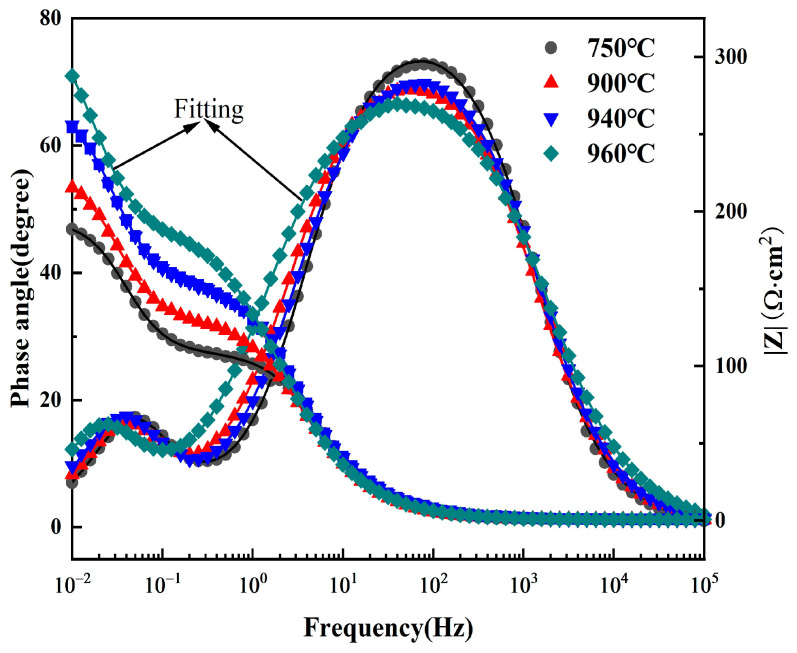
Bode diagrams of Ti90 alloy treated at different solution temperatures in 5 M HCl solution.

Figure 7 presents the Bode plot obtained in 5 M HCl solution. The Bode-phase plot shows two peaks, and the Bode-magnitude plot exhibits two steps, confirming the existence of two time constants [30]. Furthermore, the variation in low-frequency impedance with solution treatment temperature follows the same trend as observed in the Nyquist plots, indicating consistent corrosion resistance behavior across different characterization methods.

**Figure 8:**
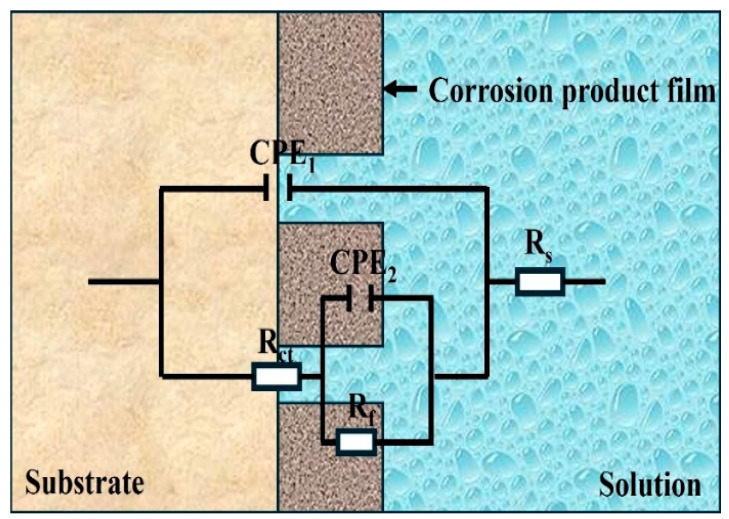
The equivalent circuits used to fit the measured impedance data.

An equivalent electronic circuit (EEC) model, as shown in Figure 8, was designed to analyze the EIS results. In the EEC, *R_S_* represents the solution resistance, *CPE_dl_* and *R_ct_*, respectively, represent the double-layer capacitance and charge transfer resistance, and *CPE_f_* and *R_f_*, respectively, represent the oxidation/corrosion product film capacitance and oxidation/corrosion product film resistance. As the electrode surface cannot reach the ideal flat state, *CPE* is adopted instead of pure capacitance [31,32]. The electrical impedance of *CPE* can be computed as follows:

(1) $$Z\left(\omega \right)={\left[Z_{0}{\left(i\omega \right)}^{n}\right]}^{-1}$$

where *ω* represents the angular frequency, *Z*_0_ represents the admittance magnitude of the CPE, *i* represents the imaginary number, and *n* represents the *CPE* exponent, which changes from 0 to 1. The detailed fitting results of EIS are listed in Table 3.

Polarization resistance (*R_p_*) provides a measure of the material’s corrosion resistance: a higher *R_p_* indicates better corrosion resistance [33]. Rp can be calculated using the following relationship [34]: *R_p_* = *R_f_* + *R_ct_*. The calculation results of the polarization resistance *R*_p_ of Ti90 alloy treated at different solution temperatures in 5 M HCl solution are shown in Table 3. As the solution treatment temperature increases, the polarization resistance (*R_p_*) of Ti90 alloy gradually increases, indicating that its corrosion resistance in 5 M HCl solution is gradually enhanced. This is also consistent with the results of the polarization curve and open-circuit potential test.

The double-layer capacitor (C_dl_) can be derived from *CPE_dl_* using Brug’s formula [35]:

(2) $$c={{CPE}_{dl}}^{\frac{1}{n}}{\left({R_{s}}^{-1}+{R_{ct}}^{-1}\right)}^{\frac{n-1}{n}}$$

*C*_dl_ and *C*_f_ were calculated by Formula (2) from the known *CPE*_dl_ and *CPE*_f_, and the calculation results are shown in Table 3. It is worth noting that the *n*_f_ index of the samples treated at different solution temperatures is very close to 1, indicating that the corrosion product film is more likely to obtain pure capacitance and has fewer defects. In the model shown in Figure 8, capacitor *C*_f_ corresponds to the capacitance in the corrosion product film. Therefore, capacitor *C*_f_ is used to calculate the thickness of the corrosion product film. The thickness of the corrosion product film can be calculated by Formula (3) [36]:

(3) $$d=\frac{\varepsilon \varepsilon_{0}}{c}$$

where ε stands the relative permittivity of the oxide layer, and *ε*_0_ represents the permittivity of free space (8.8542 × 10^−14^ F·cm^−1^). In this study, the assumed *ε* value is 65, which corresponds to the permittivity of rutile titanium dioxide [37]. The thickness of the corrosion product film is shown in Table 3. The results indicate that the thickness of the corrosion product film is relatively close, but its variation trend is inconsistent with that of the polarization resistance, suggesting that the degree of corrosion resistance is not determined by the thickness of the corrosion product film.

### 3.3. Static Immersion Test

To better analyze the corrosion resistance mechanism of Ti90 alloy with different microstructures in 5 M HCl solution, static immersion tests were performed. The temperature was maintained at 30 ± 1 °C, and the soaking duration was 7 days. The corrosion rate of Ti90 alloy was calculated from the weight loss data using Equation (4) [38]:

(4) $$\;V=\frac{KW}{AT\rho }$$

where *K* is a constant (8.76 × 10^4^), *W* is the weight loss in g, *A* is the exposed area of specimen in cm^2^, *T* is the exposure time in hours, and *ρ* is the density (4.5 g/cm^3^).

**Figure 9:**
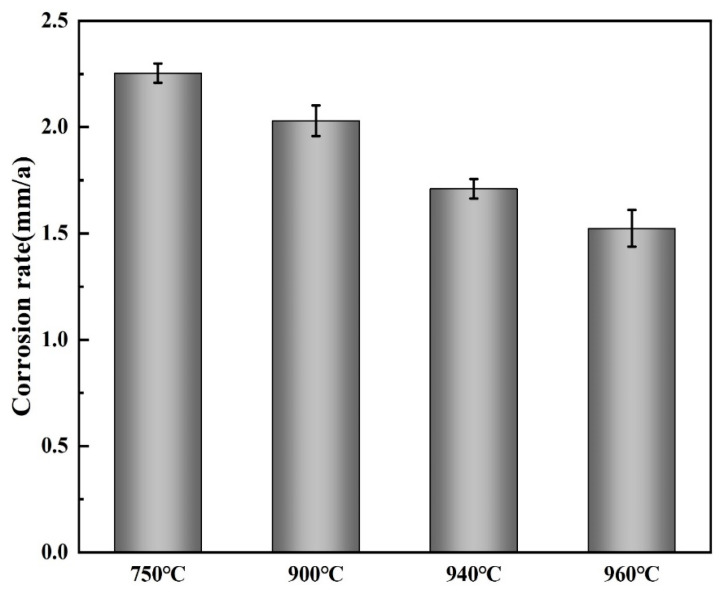
The corrosion rates of Ti90 alloy with different microstructures after being immersed in 5 M HCl solution for 7 days.

Figure 9 presents the corrosion rates of Ti90 alloy after different solution treatments. The results show that the corrosion rate in 5 M HCl solution decreases gradually with increasing solution temperature, indicating that higher solution temperatures improve the corrosion resistance of Ti90 alloy.

**Figure 10:**
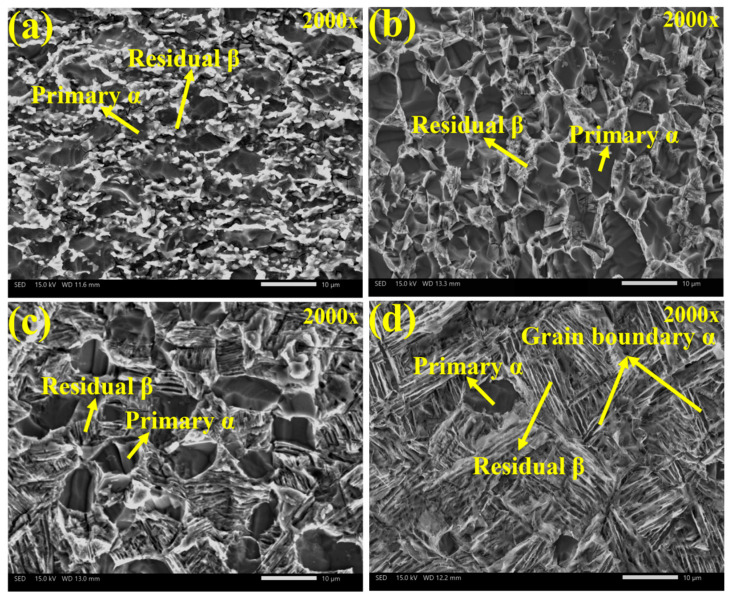
SEM morphologies of the Ti90 alloy with different microstructures in 5 M HCL solution at (**a**) 750 °C; (**b**) 900 °C; (**c**) 940 °C; and (**d**) 960 °C.

The corrosion morphology of Ti90 alloy following 7 days of immersion in 5 M HCl solution is presented in Figure 10. A comparison with Figure 2 reveals that the α phase in the microstructure corrodes preferentially and exhibits significantly more severe corrosion compared to the β-phase. As the solution treatment temperature decreases, the sample surface becomes rougher and corrosion intensifies, particularly in α phase regions. This observation aligns with the corrosion rate trend obtained from static immersion tests.

When solution-treated Ti90 alloy is immersed in 5 M HCl, its intrinsic oxide film dissolves. The OCP curves in Figure 4 clearly show an active dissolution process in 5 M HCl, corresponding to the dissolution of the primary passivation film. Figure 4 indicates that the intrinsic oxide film deteriorates after 1800 s of immersion. The chemical corrosion process in 5 M HCl occurs in two stages: degradation of the intrinsic oxide film followed by substrate dissolution. The breakdown of the native passivation film mainly involves chemical degradation of Al_2_O_3_ and TiO_2_. Upon immersion in 5 M HCl, Al_2_O_3_ first reacts with HCl as follows:

Al_2_O_3_ + 6H^+^ → 2Al^3+^ + 3H_2_O (5)

Additionally, the chemical degradation of the native oxide film is primarily due to the transformation of TiO_2_ into a hydrated state, which generally proceeds in two steps. First, TiO_2_ is reduced to TiOOH or its hydrate TiOOH·H_2_O. This reduction is balanced by oxidation of the substrate, represented by [39,40]

Ti + 3TiO_2_ + 6H_2_O → 4TiOOH·H_2_O (6)

In 5 M HCl, the high H^+^ concentration stabilizes Ti^3+^ relative to TiOOH·H_2_O, promoting reaction (7). This is consistent with the active dissolution region in the anodic polarization curve (Figure 5). The oxidation of Ti to Ti^3+^ is a major contributor to the corrosion of solid-solution Ti90 alloy:

TiOOH·H_2_O + 3H^+^ → Ti^3+^ + 3H_2_O (7)

Besides chemical dissolution, corrosion driven by the α/β micro-galvanic couple constitutes another corrosion mechanism. This micro-galvanic effect originates from elemental segregation. During micro-galvanic corrosion, the α phase acts as the anode and the β phase as the cathode, accelerating preferential dissolution of the α phase.

**Figure 11:**
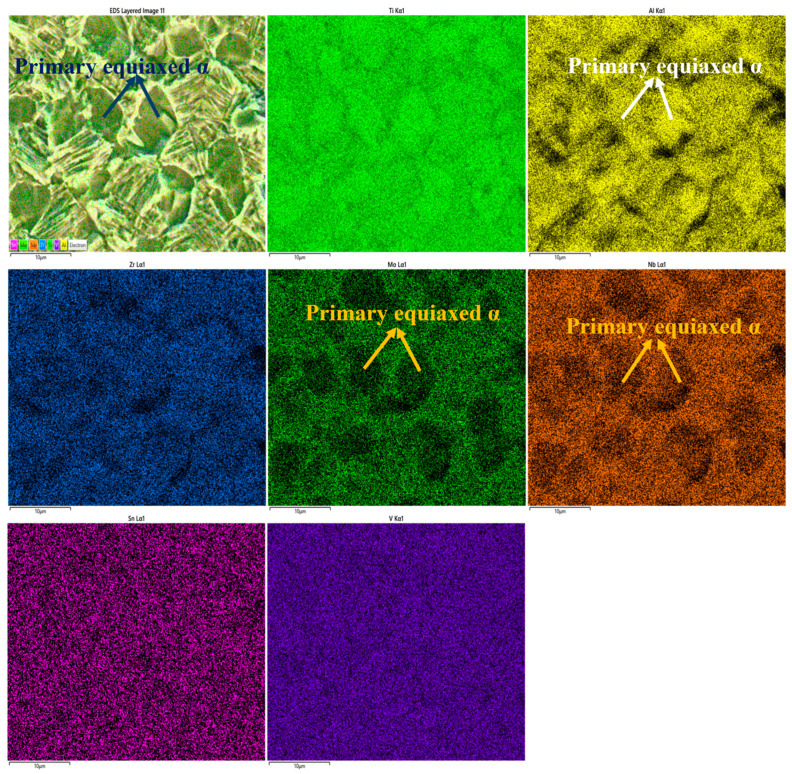
EDS result of the Ti90 alloys treated at 940 °C in 5 M HCl solution.

Previous studies [41,42] have shown that the β phase volume fraction significantly affects titanium alloy corrosion resistance. As the β phase fraction increases, the *i*_corr_ decreases, indicating improved corrosion resistance. This can be explained by the superior chemical corrosion resistance of the β phase compared to the α phase. Figure 11 shows the distribution of α and β phases on the corroded surface of solution-treated Ti90 alloy, clearly indicating the α phase as the preferred dissolution site. This preferential corrosion is attributed to elemental segregation: Al concentrates in the α phase, while Nb, Zr, Mo, Sn, and V partition to the β phase. Elements like Zr, Nb, and Mo enhance the stability of titanium alloy passivation films. Based on reactions 5–7, the corrosion of titanium alloys in HCl solution involves preferential dissolution of Al_2_O_3_ and transformation of TiO_2_ to Ti^3+^ in aqueous solution. Thus, it is reasonable to conclude that the α phase loses the protection of the intrinsic oxide film first, resulting in poorer corrosion resistance. Without this protective film, the α phase matrix is significantly more susceptible to chemical attack than the β phase.

The α phase thickness also significantly influences the corrosion resistance of solid-solution Ti90 alloy [43,44]. As shown in Figure 2, the average α phase thickness decreases with increasing solution temperature, corresponding to improved corrosion resistance in 5 M HCl. This can be explained by two factors. First, corrosion may be accelerated by micro-galvanic effects between α and β phases, arising from their different electrochemical activities due to uneven alloying element distribution. Increasing the solution temperature reduces the α phase thickness, mitigating this segregation and diminishing the micro-galvanic effect. Second, finer α phases and higher phase boundary density facilitate oxide film formation. As oxide film growth is diffusion-controlled, phase boundaries provide efficient diffusion paths for ions. The preferred diffusion of Ti, Al, Nb, Zr, Mo, and O along these boundaries supplies more oxidation and active sites for oxide film formation.

## 4. Conclusions

The microstructure and corrosion characteristics of Ti90 alloy subjected to solution treatment at different temperatures in 5 M HCl solution were investigated. The main conclusions are as follows:

(1) Microstructure analysis shows that as the solution temperature increases, the content of primary α phase decreases from 71.5% to 10.4%, and the average α phase size decreases from 2.79 μm to 0.38 μm.
(2) Potentiodynamic polarization curves reveal that all solution-treated Ti90 alloys undergo an active-to-passive transition in 5 M HCl solution. With increasing solution temperature, the *E*_corr_ shifts positively, while the *i*_corr_ decreases from 97.57 μAcm^−2^ to 38.50 μAcm^−2^, and the *i*_pass_ decreases from 97.57 μAcm^−2^ to 38.50 μAcm^−2^, indicating progressively improved corrosion resistance.
(3) EIS confirms the formation of a corrosion product film in 5 M HCl solution. The fitting results show that the polarization resistance increases from 193.2 Ω·cm2 to 317.5 Ω·cm^2^ with increasing solution temperature, further demonstrating enhanced corrosion resistance.
(4) Static immersion tests show that the corrosion rate decreases from 2.25 mm/a to 1.52 mm/a with rising solution temperature, consistent with electrochemical measurements. The α phase is found to dissolve preferentially during corrosion.
(5) The corrosion of solution-treated Ti90 alloy results from the combined effects of chemical dissolution of the primary oxide film and the substrate, as well as micro-galvanic corrosion. A higher β-phase content and a smaller average α-phase thickness in the solution-treated Ti90 alloy correspond to superior corrosion resistance.

## Figures and Tables

**Table 1:** Constituent elements of Ti90 plate (wt.%).

Ti	Al	Mo	V	Zr	Sn	Nb	O
Bal.	6.05	1.11	1.05	3.86	1.03	2.91	0.062

**Table 2:** Fitting results of potentiodynamic polarization curves.

Ti90 plates	*E*_corr_ (mV vs. Ag/AgCl)	*i*_corr_ (μA cm^−2^)	*i*_pass_ (+0.5 V) (μA cm^−2^)
750	−523.0 ± 0.51	266.86 ± 1.62	97.57 ± 0.58
900	−502.4 ± 0.36	223.19 ± 2.02	76.51 ± 0.46
940	−492.6 ± 0.43	171.69 ± 0.86	48.32 ± 0.30
960	−467.4 ± 0.58	135.69 ± 1.24	38.50 ± 0.38

**Table 3:** Fitting parameters of the equivalent circuit diagram.

Solution Temperature	*Rs* (Ω·cm^2^)	*CPE_dl_* (10^−5^ S·S^n^ cm^−2^)	*n_dl_*	*Rct* (Ω·cm^2^)	*CPE_f_* (10^−3^ S·S^n^ cm^−2^)	*n_f_*	*R_f_* (Ω·cm^2^)	*χ^2^* (10^−4^)	*λ^2^*	*R_p_* (Ω·cm^2^)	*C_dl_* (10^−5^ F cm^−2^)	*C_f_* (10^−3^ F cm^−2^)	*d* (nm)
750 °C	0.565	55.92	0.903	110.1	54.98	1	83.1	1.11	0.011	193.2	80.99	54.98	10.47
Error (%)	0.26	0.67	0.11	0.35	2.53	1.27	1.8						
900 °C	0.615	73.07	0.851	134	62.5	1	89.8	1.28	1.13	223.8	142.15	57.35	9.21
Error (%)	0.28	0.67	0.12	0.41	3.45	1.60	2.38						
940 °C	0.591	69.35	0.786	161.4	57.74	1	106	0.526	0.241	267.4	190.40	49.98	9.97
Error (%)	0.217	0.427	0.079	0.269	2.38	1.08	1.65						
960 °C	0.565	92.27	0.77	196.1	52.97	0.919	121.4	0.636	0.798	317.5	300.32	63.32	8.05
Error (%)	0.24	0.45	0.087	0.378	3.64	1.74	2.70						

## Data Availability

The original contributions presented in the study are included in the article. Further inquiries can be directed to the corresponding authors.
